# ^18^F-FDG PET/CT Findings of Non-Hodgkin Lymphoma Involving the Whole Genitourinary System

**DOI:** 10.4274/mirt.63497

**Published:** 2018-10-09

**Authors:** Aylin Oral, Bülent Yazıcı, Özgür Ömür

**Affiliations:** 1 Ege University Faculty of Medicine, Department of Nuclear Medicine, İzmir, Turkey

**Keywords:** Non-Hodgkin, extranodal, lymphoma, ^18^F-FDG, PET/CT, genitourinary system

## Abstract

A sixty-two-year-old male patient underwent orchiectomy and was diagnosed with diffuse large B-cell lymphoma in the testicle and spermatic cord. ^18^F-FDG positron emission tomography/computed tomography (PET/CT) scanning was performed for initial staging. ^18^F-FDG PET/CT scan revealed multiple hyper-metabolic lymphadenopathies, lung lesions and mass lesions in the adrenal glands and kidneys. In addition, diffuse increased ^18^F-FDG uptake suggesting lymphomatous infiltration on the right testicle, prostate and left testicular veins were detected. The genitourinary system involvement is extremely rare in extra-nodal lymphomas and to the best of our knowledge this is the first case in the literature having ^18^F-FDG accumulating lesions in all genitourinary system structures.

## Abstract (tr)

Altmış iki yaşındaki erkek hasta, uygulanan orşiektomi sonucunda testis ve spermatik kordda diffüz büyük B-hücreli lenfoma tanısı aldı. Hastaya başlangıç evrelemesi amacıyla ^18^F-FDG pozitron emisyon tomografisi/bilgisayarlı tomografi (PET/BT) tetkiki yapıldı. Yapılan ^18^F-FDG PET/BT tetkikinde, hipermetabolik özellikte çok sayıda lenfadenopatiler, akciğer lezyonları, böbreklerde ve sürrenal bezlerde kitle lezyonları saptandı. Ek olarak, sağ testiste, prostat bezinde ve sol testiküler vende lenfoma tutulumu ile uyumlu diffüz özellikte artmış ^18^F-FDG tutuluşu izlendi. Lenfomada ürogenital sistem nadir ekstranodal tutulum yerlerinden biri olup bu olgu bildiğimiz kadarıyla tüm ürogenital sistem yapılarında artmış ^18^F-FDG tutulumu gösteren lezyonlar izlenen literatürdeki ilk olgudur.

## Figures and Tables

**Figure 1:**
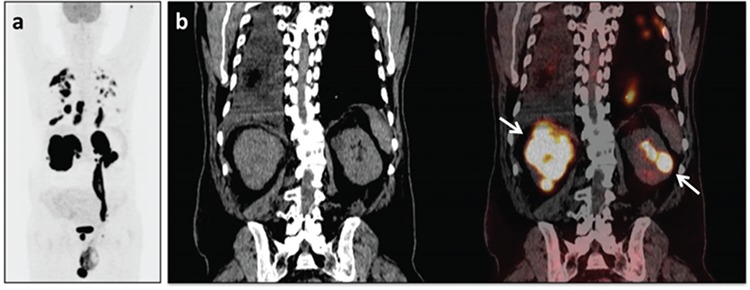
A sixty-two-year-old male patient suffering from swelling of the left testicle underwent orchiectomy and was diagnosed with diffuse large B-cell lymphoma in the testicle and spermatic cord. A) Maximum intensity projection image of the staging-intended ^18^F-FDG positron-emission tomography/computed tomography (PET/CT) scan of the case revealed multiple hyper-metabolic lymphadenopathies in the cervical, thoracic and abdominopelvic regions (SUV_max_: 34.8), hyper-metabolic lesions consistent with lymphatic/parenchymal infiltration in the lungs (SUV_max_: 16.4-45.2), pleural involvements accompanied by rib invasion on the right thorax (SUV_max_: 29.2), along with lymphoma infiltration of bilateral adrenal glands (SUV_max_: 38.7-56.5), the kidneys (SUV_max_: 40-53), the prostate gland (SUV_max_: 40.4), the right testicle (SUV_max_: 20.8) and the left testicular vein. B) Mass lesions in the kidneys are seen in selected coronal CT and fused PET/CT images.

**Figure 2:**
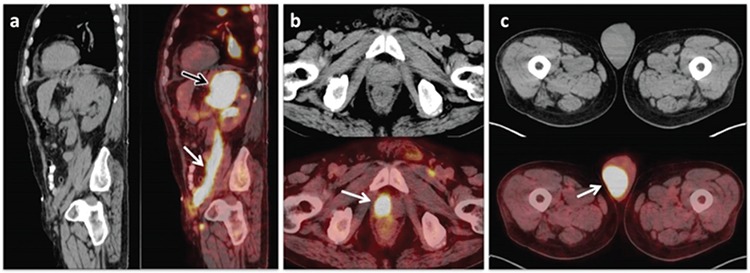
At sagittal CT and fused PET/CT images (A); mass lesion at the left adrenal gland (A; black arrow) and intense ^18^F-FDG uptake (SUV_max_: 36.8) along the left testicular vein (A; white arrow) are observed. At transaxial CT and fused PET/CT images (B, C); diffuse increased ^18^F-FDG uptake without size or density changes at CT image suggesting diffuse lymphomatous infiltration on prostate (SUV_max_: 40.4) (B) and right testicle (SUV_max_: 20.8) (C) are also seen. Although lymphomas generally originate from lymph nodes or lymphoid tissue, extranodal sites can also be involved. Extranodal lymphomas can arise in almost every organ. The most common extranodal involvement sites are gastrointestinal system, central nervous system, skin, Waldeyer’s ring, spleen and more rarely kidneys, testicle, female genital organs and liver (1,2). The genitourinary system is an extremely rare extranodal infiltration area in lymphomas, the frequency is %1-2 for testicle and less than %1 for kidney, and the dominant histological subtype is diffuse B-cell lymphoma (1,3). To the best of our knowledge this is the first case in the literature having ^18^F-FDG accumulating lesions on all of the structures of the genitourinary system. CT, the most common imaging modality at diagnosis and follow-up, is based on determining the size and the shape of lymphomatous lesions and their interface with adjacent structures. Identification of disease in normal-sized organs is difficult by anatomical imaging modalities. At this point the usefulness of the functional information provided by ^18^F-FDG PET/CT comes forward (2,4,5). For the last decades, ^18^F-FDG PET/CT has been widely used for disease staging, recurrence detection, and monitoring treatment response in patients with Hodgkin’s disease and non-Hodgkin lymphoma (4,5,6,7). ^18^F-FDG PET/CT with unenhanced CT is more sensitive and specific than contrast-enhanced CT for the detection of extralymphatic lymphomatous involvement (7). Also, ^18^F-FDG PET/CT is superior to CT for determining diffuse lymphomatous infiltration in organs (8). In this case presentation, despite urinary excretion of ^18^F-FDG, it is verified that ^18^F-FDG PET/CT is superior to CT in determining genitourinary system involvement of lymphoma, especially in diffuse lymphomatous infiltration.
